# Supplementary material for: A whole-animal phenotypic drug screen identifies suppressors of atherogenic lipoproteins
Source: eLife. 2026 Aug 27;14:RP105314. doi: 10.7554/eLife.105314 (PMC13521566; doi:10.7554/eLife.105314)
Supplement: Supplementary file 2 [file elife-105314-code1.zip › JHDL_primary_result/Danazol/Danazol_JHDL_35_026_071_2020_01_31_12_45.pdf]

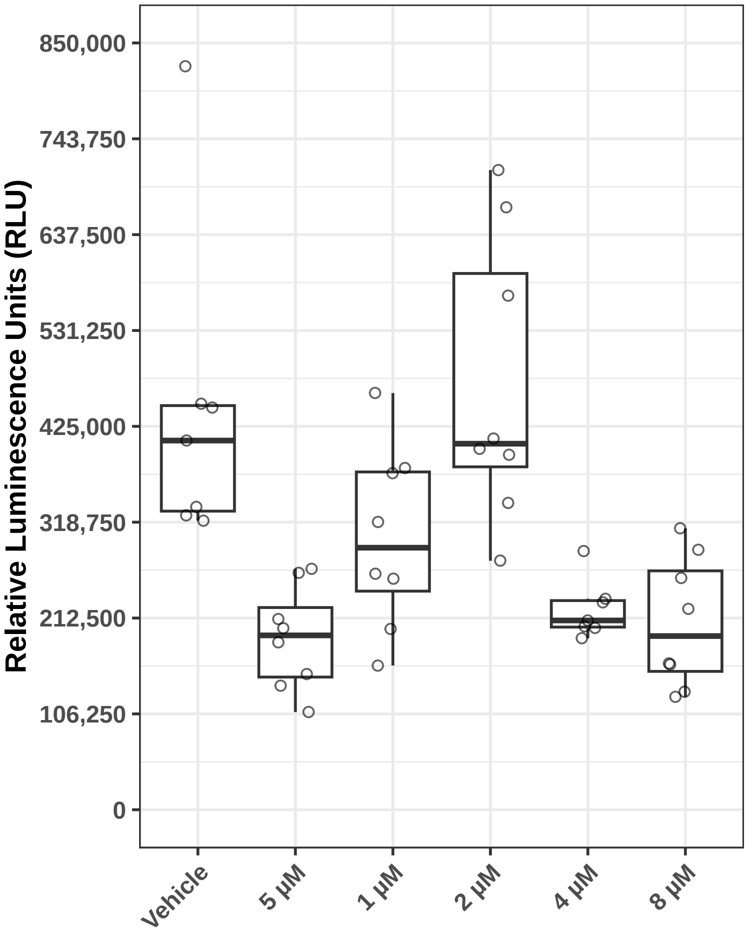

Relative Luminescence Units (RLU)
850,000
743,750
637,500
531,250
425,000
318,750
212,500
106,250
0
Vehicle
5 µM
1 µM
2 µM
4 µM
8 µM
